# Development of the Self-Directed *TRANSCEND Suffering* Workbook Intervention: A Population Health Psychology Approach for ‘Everyday’ Suffering

**DOI:** 10.3390/bs15040445

**Published:** 2025-03-31

**Authors:** Richard G. Cowden, Emily C. Hill, Omar S. Haque, Virág Zábó, György Purebl, Johannes H. De Kock, Tyler J. VanderWeele

**Affiliations:** 1 Human Flourishing Program, Institute for Quantitative Social Science, Harvard University, Cambridge, MA 02138, USA; 2 Department of Epidemiology, Harvard T.H. Chan School of Public Health, Boston, MA 02115, USA; 3 Institute of Preventive Medicine and Public Health, Semmelweis University, 1085 Budapest, Hungary; 4 Institute of Behavioral Sciences, Faculty of Medicine, Semmelweis University, 1089 Budapest, Hungary; 5 Department of Clinical Psychology, New Craigs Psychiatric Hospital, Inverness IV3 8NP, UK; 6 Department of Psychosocial Research, North West University, Potchefstroom 2530, South Africa

**Keywords:** intervention, flourishing, population health, self-transcendence, suffering, workbook

## Abstract

The discipline of psychology has long been interested in understanding human suffering and identifying suitable approaches for effectively managing it. Although there are many clinical models invoking different philosophical worldviews and therapeutic approaches for addressing suffering, they typically require trained professionals and, therefore, are not widely accessible to the general population. Empirical evidence suggests that even ‘everyday’ experiences of suffering in nonclinical populations can negatively impact mental health and well-being, which has ushered in calls for a population health psychology approach by developing accessible, affordable, and scalable interventions that attend to the experience of suffering. As a response to such calls, we developed the *TRANSCEND Suffering* workbook, a brief self-directed workbook intervention for suffering. This project report describes the first phase of the workbook development process, including its scope, theoretical underpinnings, central change objectives, organization, and engagement targets. We summarize feedback that laypeople and experts spanning various academic and applied disciplines provided about the workbook and discuss how this feedback was evaluated and used to make refinements aimed at enhancing the utility of the workbook. While empirical testing is needed to determine the efficacy of the *TRANSCEND Suffering* workbook, we discuss some potential implications (along with caveats and limitations) of this low-intensity intervention for addressing population-level suffering, facilitating growth through suffering, and promoting human flourishing.

## 1. Introduction

The discipline of psychology has long been invested in understanding and identifying suitable approaches for dealing effectively with human suffering. For example, in *The Principles of Psychology,* William James described the goal of understanding the mind’s processes as both intellectual and therapeutic when he noted, “The great thing, then, in all education, is to make our nervous system our ally instead of our enemy” (James, 1890, p. 122). Similarly, in *Civilization and its Discontents*, Sigmund Freud wrote, “If we cannot remove all suffering, we can remove some, and we can mitigate some; the experience of many thousands of years has convinced us of that” (Freud, 1962, p. 33), signaling an optimism towards the potential for ameliorating suffering. Although suffering is an important clinical concern within psychology, the concept tends to be applied more colloquially than other concepts that are indexed in diagnostic classification systems (e.g., Diagnostic and Statistical Manual of Mental Disorders, 5th Edition). A relatively common way in which this occurs is when suffering is employed as a verb to describe a person’s experience of mental health conditions (e.g., ‘suffering from depression’).

However, a growing body of work suggests that the experience of suffering—understood as “an undesired experiential state, of considerable duration or intensity, involving the loss or privation of some perceived good”—is a distinct phenomenon that may not be reducible to other clinically salient forms of psychological distress (e.g., major depressive disorder, dysthymia, or other affective disorders) and is deserving of clinical attention in its own right (Cowden et al., 2022a, p. 2; see also Yager, 2021). Consider depression as one example. Major depressive disorder is a mental health condition that is formally diagnosed by qualified healthcare professionals using established diagnostic frameworks. Suffering, on the other hand, is a subjective judgment about a negative physical or affective state that can only be made by the person experiencing it (Kauppinen, 2020). Providing empirical support for a distinction between suffering and depression, research has found that those who report symptoms that meet the threshold for potential major depressive disorder tend to score worse on a wide range of well-being indicators when symptoms are co-present with moderate/severe suffering compared to when they are not (Cowden et al., 2022b). Along similar lines, suffering is often associated with worse well-being (e.g., lower meaning in life) even after statistically controlling for other indicators of distress, such as anxiety and depression symptoms (S. Ho et al., 2022). Other research has shown that suffering has the potential to degrade well-being even when mental health symptoms do not meet the threshold for a clinical diagnosis (Wilson et al., 2007). These findings indicate that suffering is more than a marker of the severity or intensity of a person’s mental health symptoms and can have important negative effects on an individual’s well-being that are independent of processes underlying clinical diagnoses. Thus, it is important for mental health professionals to attend to the experience of suffering itself (Cowden et al., 2025).

Over the years, the discipline of psychology has developed a range of clinical models that invoke philosophical (e.g., Zen Buddhism) and therapeutic (e.g., Morita Therapy) approaches to provide a framework for addressing suffering that is tied to various mental health conditions (Prochaska & Norcross, 2018). More recent examples of these models include Acceptance and Commitment Therapy (Hayes, 2005), Integrative Meaning Therapy (Wong, 2010), Meaning-Centered Psychotherapy (Breitbart & Poppito, 2014), Mindfulness-Based Stress Reduction (Kabat-Zinn, 2005), and Mindful Self-Compassion (Germer & Neff, 2019). Although these kinds of therapeutic approaches can be effective for people who receive such treatments, many need to be delivered by qualified psychological professionals who provide services to a small fraction of the population that might be in need (Kazdin & Blase, 2011; Prochaska et al., 2020). In addition, even when psychological professionals are in abundant supply, many people lack access to insurance coverage, cannot afford their services, or find it stigmatizing to seek treatment (Patel et al., 2011). In light of growing evidence from nonclinical samples that suggests even ‘everyday’ experiences of suffering have the potential to degrade mental health and well-being (Cowden et al., 2021; Cowden et al., 2022a; S. Ho et al., 2022), calls have been made to adopt a population health psychology approach to suffering by developing scalable, low-cost, and low-intensity interventions that can be accessed readily by nonclinical populations who might be experiencing suffering (Wong et al., 2022).

A population health psychology approach to suffering would help to overcome common barriers to accessing psychological services (e.g., provider shortages, cost of treatment, concerns about stigma), particularly in less developed contexts where many of these challenges tend to be more pervasive (Dodge et al., 2024; Patel et al., 2011; Schleider et al., 2020). Because ‘everyday’ experiences of suffering in the general population are common, and the negative effects of suffering on mental health and well-being can be quite substantial, interventions that produce even modest benefits at the individual level may have a considerable impact on population-level suffering if they can be widely disseminated and used (Laynard et al., 2007; Matthay et al., 2021).

This project report describes the development of the *TRANSCEND Suffering* workbook, a brief self-directed workbook intervention for suffering that can be employed flexibly in clinical and nonclinical settings (e.g., as an ad hoc resource in formal treatment or a self-help tool), may be completed in different modes (e.g., electronically, printed out), and has the potential to be disseminated widely at low cost. We begin by describing the workbook development process, including its scope, theoretical underpinnings, central change objectives, organization, and the engagement targets tied to each component of the workbook. Next, we summarize feedback that laypeople and experts from a range of disciplines provided about the workbook, and we discuss how this feedback was evaluated and used to make refinements to enhance the utility of the workbook. We conclude with some discussion of the potential implications (along with caveats and limitations) of this low-intensity intervention for addressing population-level suffering, facilitating growth through suffering, and promoting human flourishing.

## 2. Workbook Development Process

Various models have been proposed for conceptualizing the process of developing interventions. Our approach to developing the *TRANSCEND Suffering* workbook was informed by the National Institutes of Health (NIH) Stage Model, which organizes the intervention development process in psychological science into an iterative and recursive set of six stages (see Onken et al., 2014). The workbook development process documented in this project report concerns Stage I of the model (intervention generation/refinement), focusing on activities undertaken to create the intervention (Stage IA) supplemented by some initial feasibility and pilot testing (Stage IB). By documenting this process, we intend to provide information and materials that might serve as a stepping-stone to Stage II (efficacy in research settings) or Stage III (efficacy in community settings).

We developed the *TRANSCEND Suffering* workbook to serve as a low-intensity self-directed secular intervention for adults experiencing suffering. Decisions about the scope of the workbook content and the mode of delivery were guided by our intention for the workbook to be suitable for a wide range of clinical and nonclinical adult populations, while also recognizing that (1) the workbook may not be a suitable resource for some individuals and cultural contexts, (2) the workbook is not a replacement for formal clinical treatment, and (3) clinicians (e.g., psychologists, psychiatrists, physicians) who recommend the workbook as a supplementary resource should make such decisions with care and appropriate additional supports in place. While clinicians will want to familiarize themselves with the workbook before providing it as a supplemental resource to those they work with, the workbook does not require specialized training to disseminate and can be employed flexibly.

To facilitate broad applicability and use across a diverse range of individuals, we designed the workbook with the aim of tapping into multiple modalities (e.g., psychoeducation, writing exercises, and experiential activities) so that it would engage people with different types of learning styles and preferences. For several reasons (e.g., ease of access and flexibility), we developed an editable electronic version of the workbook that is principally intended to be completed electronically via a word processor program (e.g., Microsoft Word) on a computer, tablet, or phone, or alternatively may be printed out and completed by hand. However, this editable electronic version of the workbook can easily be adapted to other modes (e.g., web-based platforms) that might provide a wider range of opportunities for access and engagement. Similar to other brief self-directed workbook interventions (e.g., *REACH Forgiveness* workbook; M. Y. Ho et al., 2024), we sought to construct a workbook requiring a nominal 3–4 h of total time to completion so that individuals might experience meaningful change in a timeframe that is both manageable and can be integrated into their daily routines.

### 2.1. Underlying Theory and Change Objectives

The *TRANSCEND Suffering* workbook is principally grounded in the paradigm of existential positive psychology (EPP; Wong, 2011), which makes several basic assumptions about suffering. Within EPP, suffering is seen as part of the ‘darker’ side of the human condition that necessarily co-exists in dialectical interplay with the ‘brighter’ side (Van Tongeren & Showalter Van Tongeren, 2021; Wong & Cowden, 2022). Since suffering is considered a universal and inescapable part of what it means to be human, it can serve an adaptive or facilitative function by pointing us to areas of our lives in need of change or providing us with an opportunity to grow in new ways. We also have the capacity to redemptively transcend and transform our suffering into the possibility of living a deeper and more fulfilling life (Frankl, 1992; Tedeschi & Calhoun, 2004; Zábó et al., in press). EPP proposes that our best defense against experiences of suffering is to approach one’s suffering with a posture of acceptance, embrace one’s suffering as a potential gateway to transformation, and seek to transcend one’s suffering by responding courageously (Wong et al., 2022). It is these principles that provide the scaffolding for the overall arc of the workbook and its core change objectives.

Although the *TRANSCEND Suffering* workbook aims to lessen a person’s current experience of suffering, it seeks to achieve this indirectly by helping them to reflect on their suffering from a more balanced and holistic perspective, consider the possibility that they might be able to respond differently to their suffering, and explore ways in which they can meaningfully engage with life in the midst of their suffering. This approach is not only consistent with many therapeutic models that are commonly used in clinical settings, such as Acceptance and Commitment Therapy (Hayes, 2005), but also aligns with recent work on suffering itself. For example, Zábó et al. (in press) employ the concept of suffering mindsets to explore how a person’s beliefs about whether suffering is fundamentally debilitative versus facilitative might affect their current experience of suffering itself, how they respond to their current suffering, and the effect that their current suffering has on their well-being. More specifically, they suggest that people who believe suffering has constructive and ennobling consequences (i.e., a facilitative mindset about suffering) might be less likely to endorse suffering when they encounter a situation that has the potential to precipitate suffering. Thus, a person who experiences a facilitative shift in their suffering mindset might be inclined to reinterpret what they are experiencing in more positive terms, even if there has not been a substantial change in the actual circumstances they are facing (Beng et al., 2021; S. Ho et al., 2022). This kind of shift in mindset might also lead to more adaptive responses to suffering itself, such as when a person relinquishes their attempts to avoid their suffering and instead chooses to embrace it with courage or cope with it actively and adaptively (Zábó et al., in press). While a mindset shift about suffering is just one way that those who are suffering might benefit from the *TRANSCEND Suffering* workbook, it illustrates how the workbook could have more of an indirect (rather than direct) effect on suffering. More generally, intentionally using suffering to foster positive transformation may increase the likelihood of such transformation occurring.

### 2.2. Workbook Structure and Engagement Targets

Guided by the underlying scaffolding of the abovementioned principles from EPP, the *TRANSCEND Suffering* workbook is organized into nine components that correspond with its central change objectives: **T**urn toward your suffering; **R**eflect on the suffering you are experiencing; **A**ccept what is beyond your control; **N**otice the goodness in your life; **S**ee the possibility of transcending your suffering; **C**hoose the path you will take from this point forward; **E**stablish a self-transcendent purpose; **N**ame specific goals that align with your self-transcendent purpose; **D**ive into your self-transcendent purpose.

Each component is underpinned by two key engagement targets that guide the structure, scope, and activities used to facilitate change (see Table 1). The team involved in constructing the workbook developed the content and activities through collaborative engagement with one another by drawing on their collective expertise as theoreticians, researchers, and clinicians within the disciplines of psychology and psychiatry, supplemented in some cases by the use of existing materials (e.g., meditation activity in Exercise 4.2). Although the engagement targets guided decisions about the content and activities that form the central ingredients for each component of the workbook, to optimize flow and engagement, there may be instances in which the content or activities for a component extend beyond the specified engagement targets. The workbook draws on multiple modalities—such as written, meditative, and experiential activities (e.g., role-playing)—to promote change that aligns with the engagement targets for each component.

We sought to develop a workbook that not only shares common ground with relevant therapeutic models but also extends beyond the boundaries of any singular therapeutic model to support change. The *TRANSCEND Suffering* workbook is unique in its synthesis of, and compatibility with, a number of different therapeutic models that are frequently employed by psychological professionals in their clinical work (for a non-exhaustive list of therapeutic models that connect to different components of the workbook, see Table 1). For example, turning toward suffering aligns with mindfulness meditation and exposure therapy; reflecting on suffering is compatible with cognitive behavioral therapy; noticing the goodness in one’s life incorporates elements of gratitude practices from positive psychology; seeing the possibility of transcending your suffering includes elements of optimism practices and strengths-finding therapies in positive psychology as well as cognitive restructuring from cognitive behavioral therapy; establishing and pursuing a self-transcendent purpose coheres with meaning-oriented therapies and logotherapy; sharing lessons learned to encourage others with transcending suffering drafts from altruism interventions in positive psychology. While the workbook shares points of connection with an eclectic network of therapeutic models, it is unique in that its focus is not merely on aiming to avoid suffering, distract oneself from suffering, or eliminate suffering as quickly as possible, but rather on turning towards and working with and through suffering for transformation and growth.

### 2.3. Feedback and Refinement

Although the *TRANSCEND Suffering* workbook was developed iteratively through consensus among the interdisciplinary group of authors involved in its construction, we sought feedback from academics representing different disciplines (e.g., sociology, theology), religious leaders/practitioners, and clinical professionals (e.g., psychologists, physicians) who had subject-matter, research, and/or applied expertise concerning the topic of human suffering. We identified 13 experts through our network who were willing to provide written feedback on the workbook, which included three psychologists, three religious leaders/practitioners, two psychiatrists, two physicians, a sociologist, a theologian, and a public health expert. We also used our extended social networks to disseminate the workbook to a sociodemographically diverse group of adults in the United States (*n* = 6) and Hungary (*n* = 14) who were interested in completing the workbook with reference to their current experience of suffering and providing written feedback. Experts received guiding questions prompting them to provide feedback by reflecting on potential users of the workbook (e.g., “Is the workbook an appropriate resource for people experiencing suffering?”). The guiding questions provided to laypeople prompted them to respond by reflecting on their experience of going through the workbook (e.g., “Did you experience any challenges completing the workbook?”). Guiding questions were accompanied by follow-up prompts to gather additional detail and explanation. Differences in guiding questions solicited somewhat different feedback from experts and laypeople.

We synthesized the feedback received from experts and laypeople (separately) using thematic analysis to identify key themes (Braun & Clarke, 2006). Themes that emerged for the experts pointed to: (1) aspects of the language, structure, conceptual content, and/or exercises in certain parts of the workbook that could benefit from clarification (e.g., outline the flow of the workbook, provide more detailed instructions to some exercises); (2) opportunities for improving the relevance of some of the conceptual content and exercises in the workbook (e.g., reduce instances of ambiguous language or phrasing, consider using alternative exercises in some places); (3) some potential ways to enhance engagement with the workbook (e.g., offer encouragement periodically, combine exercises that appeared redundant); (4) important strengths of the workbook (e.g., use of key people and stories throughout, complex concepts made accessible); and (5) considerations for dissemination of the workbook (e.g., as a supplement to clinical treatment, target population suggestions). Themes that emerged based on the feedback received from laypeople centered on (1) aspects of the language, structure, and/or exercises that made it challenging to complete some parts of the workbook (e.g., lack of transitions between some lessons, difficulties executing certain meditations) and (2) important strengths of the workbook (e.g., reframing of suffering, use of multiple modalities). Aspects of the workbook that involved more direct engagement with present suffering (e.g., Lesson 4), required a greater investment of time (e.g., Lesson 13), and encouraged users to contact or interact with others (e.g., Lesson 2) were considered more challenging.

We attempted to incorporate all feedback that was received into the refined version of the workbook, except when feedback did not align with the core change objectives and/or engagement targets of the workbook (e.g., suggestions to remove key content). The team involved in developing the workbook collaboratively evaluated and decided on refinements aimed at strengthening the workbook. The refinements that were made can be grouped thematically into (1) clarifying and simplifying language in certain places; (2) making adjustments to points of focus and emphasis; (3) including additional examples and encouragement statements; and (4) improving aspects of the structure and organization. The type and number of refinements for components of the workbook, along with notable examples of those refinements, are summarized in Table 2. The final Microsoft Word-compatible (English) version of the *TRANSCEND Suffering* workbook can be freely accessed and downloaded via https://osf.io/f7wm9/ (see Supplementary Materials for a PDF version).

## 3. Discussion

The *TRANSCEND Suffering* workbook is a response to recent calls that point to the need for low-intensity, population-level interventions that could be accessed readily and disseminated widely to support people who are suffering (Wong et al., 2022). Following the NIH Stage Model (Onken et al., 2014), this project report documented the foundational intervention generation process that was undertaken as part of Stage I. Before investing resources to disseminate the *TRANSCEND Suffering* workbook at scale, its efficacy must first be evaluated in different research and/or community settings (Stage II and/or III).

### 3.1. Anticipated Effects and Possible Mechanisms

To our knowledge, this is the first self-directed workbook intervention for suffering that draws on principles of EPP, a paradigm that puts forward a somewhat unique theoretical position that the ‘dark’ experience of suffering ought to be considered alongside the ‘bright’ possibility of transformation and growth through suffering (Wong & Cowden, 2022). Although different forms of transformation or growth might be possible through suffering, the *TRANSCEND Suffering* workbook is principally intended to stimulate an epistemic form of growth characterized by a change in a person’s fundamental beliefs (or mindset) about suffering that enables them to consider suffering in a more balanced and holistic way. Zábó et al. (in press) theorize that this kind of shift in a person’s mindset about suffering may lower their perception of suffering, even if the objective features of their situation have not changed, because the lens through which they are interpreting their experience has shifted. This suggests that a key mechanism underlying change in suffering might be a positive shift in mindset about suffering. In addition, a positive shift in mindset about suffering might be involved in catalyzing improvements in other outcomes beyond suffering, in part because empirical evidence suggests people often experience improvements in various facets of well-being (e.g., life satisfaction, meaning in life) when their suffering diminishes (Cowden et al., 2021; S. Ho et al., 2022). If engagement with the *TRANSCEND Suffering* workbook effectively reduces a person’s suffering, then it is not unreasonable to expect that positive changes in well-being might also be observed.

While a shift in a person’s mindset about suffering might be a central mechanism underlying change that is facilitated by the *TRANSCEND Suffering* workbook, the intervention takes an eclectic approach that shares points of connection to many different theories, therapeutic models, and modes of engagement. Therefore, it is possible that a combination of mechanisms might be operative. For example, reflecting on and writing about people or experiences that one is grateful for may provide some relief from suffering by broadening a person’s perspective beyond the negative features of their situation and eliciting positive emotions (Cregg & Cheavens, 2021; Emmons & Stern, 2013), whereas the process of evaluating personal strengths and social resources that helped with past suffering might diminish present suffering by building courage and confidence in the possibility of transcending one’s current circumstances (Hannah et al., 2010; McAdams et al., 2001). By specifying engagement targets per component of the workbook and attempting to align the content and activities of each component with those targets, we expect the workbook development process outlined in this paper will provide opportunities to understand the mechanisms of change that might be operative and how those mechanisms are distributed across different components of the workbook.

### 3.2. Flexibility and Scaling Considerations

**Figure 1:**
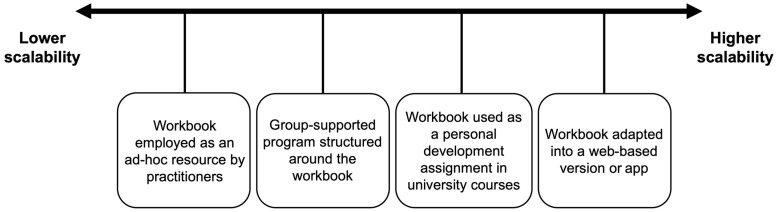
Examples of dissemination possibilities for the *TRANSCEND Suffering* workbook intervention.

Because the *TRANSCEND Suffering* workbook is a self-directed intervention that circumvents a number of barriers known to impinge on access to psychological services (e.g., treatment stigma, cost of treatment), its dissemination potential is high and could be employed flexibly at varying degrees of scale (see Figure 1). At a more individual level, the workbook could be used by practitioners as an ad hoc resource to support some of the people they work with. A slightly more scalable approach might be to use the workbook as the foundation for a group-supported program in which individual completion of the workbook is intermingled with group-based meetings guided by a suitable facilitator. Moving higher up on the scalability spectrum, the workbook could be integrated into relevant university-level courses as a personal development assignment. Finally, the contents of the workbook could be programmed into a free or low-cost web-based version or app that automatically delivers the content through reminders or even an artificial intelligence-based chatbot. These are merely illustrative examples that demonstrate the flexibility of the workbook and possibilities for dissemination.

Although key strengths of this self-directed workbook model are scalability and flexibility, it is worth considering the trade-off between scalability and the extent of intervention uptake. A highly scalable intervention may not necessarily result in high uptake even if its reach is expansive, highlighting the need for effective communication and engagement strategies to accompany attempts at scaling the *TRANSCEND Suffering* workbook as a public health intervention, such as through facilitators, coaches, clinicians, teachers, and/or social workers. A combination of dissemination approaches that vary in scalability may be needed to optimize the impact of the workbook as a population health psychology approach to supporting people who are suffering.

### 3.3. Caveats and Limitations

Although suffering is a shared human experience, it is often experienced very differently across individuals (Cassell, 2004; Tate & Pearlman, 2019). We expect there to be some variability both in terms of the extent to which individuals might benefit from the *TRANSCEND Suffering* workbook as well as how they might benefit. For instance, the workbook might not be a fitting resource for people who are experiencing suffering near the end of life because of a terminal illness, whereas it may be a helpful resource for those whose suffering is tied to a chronic condition that can affect quality of life (e.g., pain, major depressive disorder). Future research could explore potential factors (e.g., individual differences, developmental stages, cultural variation) that contribute to variability in the effects of the workbook to identify which population segments are more vs. less likely to benefit from it.

The *TRANSCEND Suffering* workbook was designed with the intention of having broad population-level utility, but it may not be an appropriate resource for all individuals who are experiencing suffering. For example, it would not be suitable for acute stress responses or in the immediate aftermath of traumatic and life-threatening situations. In addition, the workbook format is not likely to be the most suitable resource for people to complete independently if they have a physical disability that restricts them from being able to use electronic devices or printed materials. Further work is needed to explore possibilities of adapting the workbook into other modes of delivery (e.g., an audio-based app version) that might be able to address some of the limits of the workbook format.

We have developed the *TRANSCEND Suffering* workbook so that it can be accessible to and used by people from different worldviews, religious/spiritual backgrounds, and cultures. While the workbook does draw upon insights and certain assumptions found within EPP (Wong, 2011), it is not tied to a specific religious tradition. In many ways, this can be seen as a strength of the workbook concerning its potential wide applicability. Nevertheless, many of the world religions do themselves have teachings on the nature of suffering, the meaning of suffering, and the seeking of transformation through suffering, and the understanding of suffering across these traditions certainly does vary (Fitzpatrick et al., 2016; Scheler, 1992). While we believe the *TRANSCEND Suffering* workbook would be of value across many world religions, resources that are particularly adapted to ideas on suffering within a specific religious tradition might be especially effective for those within that tradition. Religious and cultural adaptation or modification of the *TRANSCEND Suffering* workbook would constitute an interesting and important direction for future development and research.

We have proposed that the *TRANSCEND Suffering* workbook may serve as a useful ad hoc resource that practitioners who are working in various clinical roles (e.g., psychologists, physicians) could offer to clients/patients. While much of the feedback that we received from professionals working in clinical roles aligns with this view, appropriate clinical judgement must be used when making decisions along these lines. Even when clinical judgement suggests that offering the workbook to a client/patient could be complementary to the treatment approach, some clients/patients may benefit from integrating their experience of engaging with the workbook into the treatment process.

While the self-directed format of the *TRANSCEND Suffering* workbook has its advantages (e.g., broad accessibility at low cost), self-directed interventions also have potential drawbacks that can impact their effectiveness. For example, self-directed interventions tend to offer limited personalization and real-time feedback, which can be valuable for supporting progress (Garrido et al., 2019). Without guidance and encouragement, some users may struggle to stay engaged and adhere fully to the intervention (Baumeister et al., 2014). For those experiencing more severe suffering, the absence of professional supervision during the process of working through self-guided materials could increase the risk of adverse outcomes (Papworth et al., 2015). Some of these (and other) potential drawbacks of self-directed interventions may also apply to the *TRANSCEND Suffering* workbook.

## 4. Conclusions

This project report described the initial phase of developing the *TRANSCEND Suffering* workbook, a theoretically and evidence-informed self-directed intervention. While rigorous studies (e.g., randomized controlled trials) are needed to evaluate the efficacy of this novel low-intensity intervention to address suffering and promote growth through suffering in different populations and cultural contexts, there is considerable potential for the *TRANSCEND Suffering* workbook to serve as a complementary resource in clinical settings and an accessible standalone resource for nonclinical populations. By documenting the process of developing the *TRANSCEND Suffering* workbook and offering the intervention as a freely available resource, we hope that our approach will encourage others to consider adopting a population health psychology lens to promote human flourishing.

## Figures and Tables

**Table 1:** *TRANSCEND Suffering* workbook components, engagement targets, and connecting theories and clinical models.

Components	Engagement Targets	Connecting Theories ^1^	Connecting Clinical Models ^1^
**T**urn toward your suffering	Develop a deeper understanding of their own ‘suffering mindset’ Express the suffering they are presently experiencing	Existential positive psychology (Wong et al., 2022) Humanistic psychology (Buhler, 1971) Meaning making model (Park, 2010) Narrative psychology (Sarbin, 1986) Positive psychology (Seligman & Csikszentmihalyi, 2000)	Acceptance and commitment therapy (Hayes, 2005) Dialectical behavior therapy (Linehan & Wilks, 2015) Existential therapy (van Deurzen-Smith, 1988) Meaning-centered counseling and therapy (Wong, 2013) Narrative therapy (White & Epston, 1990)
**R**eflect on the suffering you are experiencing	Gain further insight into the source/s of their present suffering Identify the impact/s of their present suffering on different dimensions of their life		
**A**ccept what is beyond your control	Identify the aspect/s of their present experience that are within their personal control and those that are not Experience an increased sense of acceptance for the aspect/s of their present suffering that are outside their personal control		
**N**otice the goodness in your life	Experience increased gratitude for important people that have been part of their life Experience increased gratitude for specific events and/or circumstances that have occurred during their life		
**S**ee the possibility of transcending your suffering	Connect with personal strengths and social resources that helped them overcome past suffering Identify personal strengths they can draw on as they navigate their present suffering		
**C**hoose the path you will take from this point forward	Gain further insight into their capacity to exercise personal agency even in the midst of suffering Identify areas in which they plan to exercise personal agency in order to make changes in areas that are within their personal control		
**E**stablish a self-transcendent purpose	Understand more fully what it means to have a self-transcendent purpose Develop a personalized self-transcendent purpose statement		
**N**ame specific goals that align with your self-transcendent purpose	Construct specific short-term and longer-term goals that align with their self-transcendent purpose Establish a timeline for evaluating progress against specific short-term and longer-term goals		
**D**ive into your self-transcendent purpose	Make a formal commitment to live out their self-transcendent purpose Compile a testimony of their journey through the workbook and shares lessons learned to encourage others with transcending suffering		

Note. ^1^ Lists for connecting theories and clinical models are intended to be representative rather than exhaustive.

**Table 2:** Summary of revisions to the *TRANSCEND Suffering* workbook.

Revision Type	Component Affected (Lesson and Number of Revisions)	Examples of Revisions
Clarifications and simplification to language	Turn toward your suffering (Lesson 2: eleven revisions) Reflect on the suffering you are experiencing (Lesson 3: five revisions) Accept what is beyond your control (Lesson 5: two revisions; Lesson 6: one revision) Notice the goodness in your life (Lesson 7: three revisions) See the possibility of transcending your suffering (Lesson 8: four revisions; Lesson 9: one revision) Choose the path you will take from this point forward (Lesson 11: one revision) Establish a self-transcendent purpose (Lesson 13: ten revisions; Lesson 14: sixteen revisions) Name specific goals that align with your self-transcendent purpose (Lesson 15: twenty revisions; Lesson 16: four revisions) Dive into your self-transcendent purpose (Lesson 17: twelve revisions; Lesson 18: three revisions)	Removed unnecessary detail, such as background information about Boethius’ translation work (Lesson 2) Removed or replaced jargon, such as “microcosm” (Lesson 7) Changed lesson title to align better with engagement targets (Lesson 8) Changed the post-meditation writing prompt (Lesson 9)
Adjustments to focus and emphasis	Turn toward your suffering (Lesson 2: two revisions) Reflect on the suffering you are experiencing (Lesson 3: seven revisions; Lesson 4: one revision) Accept what is beyond your control (Lesson 6: two revisions) Notice the goodness in your life (Lesson 7: one revision) See the possibility of transcending your suffering (Lesson 9: one revision) Establish a self-transcendent purpose (Lesson 13: seven revisions; Lesson 14: three revisions) Name specific goals that align with your self-transcendent purpose (Lesson 15: one revision; Lesson 16: one revision)	Added language to more effectively use the story of Boethius to illustrate how a person can change how they relate to suffering even when they are unable to control its causes (Lesson 6) Refined meditation (Lesson 9) Added a paragraph expressing that making choices which lead to overcoming suffering is something we must do repeatedly (Lesson 11) De-emphasized the necessity of finding a lifelong purpose (Lesson 13)
Addition of examples and encouragement statements	Turn toward your suffering (Lesson 2: three revisions) Reflect on the suffering you are experiencing (Lesson 3: three revisions; Lesson 4: one revision) Accept what is beyond your control (Lesson 5: one revision; Lesson 6: two revisions) Notice the goodness in your life (Lesson 7: two revisions) See the possibility of transcending your suffering (Lesson 8: four revisions; Lesson 9: two revisions) Establish a self-transcendent purpose (Lesson 13: one revision) Name specific goals that align with your self-transcendent purpose (Lesson 15: one revision; Lesson 16: one revision)	Added a somatic meditation before the first writing reflection activity (Lesson 2) Added examples of internal and external sources of suffering (Lesson 3) Added a sentence acknowledging progress made so far (Lesson 5) Added examples for the gratitude exercise (Lesson 7)
Improvements to structure and organization	Turn toward your suffering (Lesson 1: one revision; Lesson 2: one revision) Accept what is beyond your control (Lesson 5: one revision; Lesson 6: one revision) Establish a self-transcendent purpose (Lesson 13: one revision; Lesson 14: one revision) Dive into your self-transcendent purpose (Lesson 18: two revisions)	Changed the order of question prompts to build towards more open-ended questions (Lesson 1) Replaced a repetitive exercise with a meditation (Lesson 6) Moved a section from Lesson 14 to Lesson 13 Moved a paragraph explaining why it is important to share personal testimony so that it comes before the exercise (Lesson 18)

## Data Availability

Data is contained within the article and Supplementary Materials.

## Supplementary Materials

The following supporting information can be downloaded at: https://www.mdpi.com/article/10.3390/bs15040445/s1, A Workbook to TRANSCEND Suffering. References (Boethius, 1999; Campbell, 1949; Neff, 2022) are cited within the Supplementary Materials.
